# Mitigating multiple stresses in *Pangasianodon hypophthalmus* with a novel dietary mixture of selenium nanoparticles and Omega-3-fatty acid

**DOI:** 10.1038/s41598-021-98582-9

**Published:** 2021-09-30

**Authors:** Neeraj Kumar, Dilip Kumar Singh, Shashi Bhushan, Ankur Jamwal

**Affiliations:** 1 ICAR-National Institute of Abiotic Stress Management, Baramati, Pune, Maharashtra 413115 India; 2 ICAR-Central Institute of Fisheries Education, Versova, Mumbai, 400061 India; 3 DRPCAU-College of Fisheries Dholi, Samastipur, Bihar 848125 India

**Keywords:** Zoology, Ichthyology

## Abstract

Effects of a novel dietary mixture of selenium nanoparticles (Se-NPs) and omega-3-fatty acids i.e., Eicosapentaenoic acid (EPA) and docosahexaenoic acid (DHA) on mitigating arsenic pollution, high-temperature stress and bacterial infection were investigated in *Pangasianodon hypophthalmus*. To aim this, four isocaloric and iso-nitrogenous diets were prepared: control feed (no supplementation), Se-NPs at 0.2 mg kg^−1^ diet with EPA + DHA at 0.2, 0.4 and 0.6% as supplemented diets. Fish were reared under normal condition or concurrent exposure to arsenic (2.65 mg L^−1^), and temperature (34 °C) (As + T) stress for 105 days. The experiment was conducted with eight treatments in triplicates. Response to various stresses i.e., primary (cortisol), secondary (oxidative stress, immunity, and stress biomarkers) and tertiary stress response (growth performance, bioaccumulation and mortality due to bacterial infection) were determined. Supplementation of dietary Se-NPs at 0.2 mg kg^−1^ diet and EPA + DHA at 0.2 and 0.4% reduced the primary stress level. Exposure to arsenic and temperature (As + T) and fed with control diet and EPA + DHA at 0.6% aggravated the cortisol level. Anti-oxidative enzymes (Catalase, superoxide dismutase, glutathione peroxidase and glutathione-s-transferase) and immunity (Nitroblue tetrazolium, total protein, albumin, globulin, A:G ratio, total immunoglobulin and myeloperoxidase) of the fish were augmented by supplementation of Se-NPs and EPA + DHA at 0.2 and 0.4%. Neurotransmitter enzyme, HSP 70, Vitamin C were significantly enhanced (p < 0.01) with supplementation of Se-NPs at 0.2 mg kg^−1^ and EPA + DHA at 0.2 and 0.4%. Whereas total lipid, cholesterol, phospholipid, triglyceride and very low-density lipoprotein (VLDL) were reduced (p < 0.01) with the supplementation of Se-NPs at 0.2 mg kg^−1^ diet and EPA + DHA at 0.2 and 0.4%. Tertiary stress response viz. growth performance was also significantly enhanced with supplementation of Se-NPs at 0.2 mg kg^−1^ and EPA + DHA at 0.2 and 0.4% reared under As + T. Whereas arsenic bioaccumulation in fish tissues was significantly reduced with dietary supplementation of Se-NPs and EPA + DHA. Cumulative mortality and relative percentage survival were reduced with Se-NPs at 0.2 mg kg^−1^ and EPA + DHA at 0.2 and 0.4%. The investigation revealed that a novel combination of Se-NPs at 0.2 mg kg^−1^ and EPA + DHA at 0.4% followed by 0.2% has the potential to alleviate temperature stress, bacterial infection and arsenic pollution. Whereas diet containing Se-NPs at 0.2 mg kg^−1^ diet and EPA + DHA at 0.6% was noticeably enhanced the stress in *P. hypophthalmus*.

## Introduction

Climate change and pollution are the major challenges affecting the functioning of ecosystems worldwide for last two decades^1^. Global warming has been one of the contributing factors for arsenic contamination of food and water, especially in Asian countries dependent on groundwater withdrawals^2^. It is now established that chronic exposure to water and foodborne arsenic can cause adverse health effects, including cancer, in humans. Since the North-Eastern states of India have high arsenite content in groundwater, crops and fish, which may accumulate arsenic from water and cause arsenicosis (toxicity from chronic exposure to arsenic) in humans^3–5^. Arsenic can contaminate ecosystems through natural as well as anthropogenic activity^6^. In addition to the toxic effects of arsenic on human a higher concentration of arsenic in aquatic systems can also deteriorate the condition of aquatic animals including fishes^7,8^. Arsenite binds with sulfhydryl groups of biomolecules viz. glutathione (GSH), lipoic acid and the cysteinyl residues of many enzymes, causing toxicity^9^.

Eicosapentaenoic acid (EPA, 20:5n-3) and docosahexaenoic acid (DHA, 22:6n-3) are strong antioxidants and essential fatty acid (EFAs) which belong to omega-3-fatty acid. Linolenic (18:3n-3) and linoleic acid (18:2n-6) are the precursor for synthesis of EPA and DHA which has been absent in the fish^10^. The omega-3 fatty acids play essential roles in a wide variety of physiological functions, such as neural and visual system^11,12^, pigmentation^13,14^, bone development^15,16^, reproduction^17^, stress resistance^18,19^ and immunity^20,21^ as well as growth and development of the fish^22,23^, and anxiety-like behaviour and emotions^24,25^ in animals. Despite the essential roles of EPA and DHA in physiology, fish have a negligible capacity to synthesize them, thus necessitating dietary intake^10,26^. Selenium (Se) is an essential trace element for growth, immunity and maintains redox homeostasis through selenoproteins such as the glutathione peroxidase, which reduces hydrogen peroxide through the glutathione-mediated pathway in fish^27,28^. Selenium dependent glutathione peroxidase is very useful to control the redox state in the cell. Other selenoenzymes, viz. methionine sulfoxide reductase and thioredoxin reductase also maintain redox homeostasis and prevent oxidative damage of lipids and other biomolecules by terminating redox-reactive radicals^29,30^. Furthermore, selenoproteins have crucial roles in cell proliferation activation, cell differentiation, innate and adaptive immunity^31,32^. Selenium is also known to antagonize toxicity of elements such as cadmium and arsenite in fishes, primarily by attenuation of oxidative stress^33,34^. Our previous works have demonstrated that Se-NPs supplementation in fish diet could stimulate growth performance, immunity, anti-oxidative status and thermal tolerance^35–39^ in fish. Moreover, Se-NPs are more efficient and less toxic than the usual form of elemental selenium^40^.

Oxidative stress is the biological process in which an unpaired electron, present on reactive radical, damages the tissues in living organisms including animal and fish. These oxidizing radicals are formed as by-products of aerobic respiration^41^. Oxidizing radicals could exist as free radicals such as (OH^·^), superoxide (O2^·−^), nitric oxide (NO^·^), nitrogen dioxide (NO2^·^), peroxyl (ROO^·^) and lipid peroxyl (LOO^·^) and non-free radicals hydrogen peroxide (H_2_O_2_), ozone (O_3_), singlet oxygen (^1^O_2_), hypochlorous acid (HOCl), nitrous acid (HNO_2_), peroxynitrite (ONOO^−^), dinitrogen trioxide (N_2_O_3_), lipid peroxide (LOOH)^42^. Such free radicals and non-free radicals are neutralized by enzymes such as catalase (CAT), superoxide dismutase (SOD), glutathione-s-transferase (GST) and glutathione peroxidase (GPx). Therefore, a combination of Se-NPs and EPA + DHA can protect the cell against oxidative stress through enhancing anti-oxidative enzymatic systems. The mixture may also have role in improving the immunity of the fish. The present study was carried out to evaluate the protective role of dietary mixture of Se-NPs and EPA + DHA against arsenic pollution, high temperature stress and bacterial infection in *Pangasianodon hypophthalmus*.

## Material and methods

### Ethics statement

The study protocol and the end-points of the experiments were approved by the Research Advisory Committee of ICAR-NIASM. All methods were carried out in accordance with relevant national and international guidelines and regulations. The study is in compliance with the Animal Research: Reporting of In Vivo Experiments (ARRIVE) guidelines.

### Diet preparation

Four iso-nitrogenous (35% crude protein) and iso-caloric (393 kcal/100 g) experimental diets were prepared. The basal diet containing soybean meal, fish meal, groundnut meal, wheat flour, cod liver oil, carboxymethyl cellulose (CMC), lecithin, and vitamin C. Selenium-free vitamin-mineral mixture was prepared manually for inclusion in the experimental diet. The heat-labile components were incorporated after cooking the feed ingredients (Table 1). The control diet received no Se-NP or EPA + DHA supplementation. The rest of the three experimental diets contained 0.2 mg Se-NP kg^−1^ diet, in mixture with EPA + DHA at 0.2, 0.4 and 0.6%. Proximate composition of four experimental diets were analyzed using AOAC method^43^ (Table 1). Crude protein (CP) was estimated using nitrogen content and ether extract (EE) by solvent extraction method. Ash (mineral content) was estimated by completely burning the feed overnight in a muffle furnace at 550 °C. Total carbohydrate % was calculated using the following equation:

$${\text{Total}}\,{\text{carbohydrate}}\,\% = {1}00 - \left( {{\text{CP}}\% + {\text{EE}}\% + {\text{Ash }}\% } \right).$$

The gross energy of the diets were calculated by the use of the method described by Halver^44^.

**Table 1:** Composition and proximate analysis of experimental diet (% dry matter) of selenium nanoparticles (Se-NPs), eicosapentaenoic acid (EPA) and docosahexaenoic acid (DHA) fed to *Pangasianodon hypophthalmus* during the experimental period of 105 days.

Ingredient	Control diet	Selenium nanoparticles (Se-NPs) + EPA + DHA		
	Selenium and EPA + DHA free diet	0.2 mg/kg + 5 mg/kg diet	0.2 mg/kg + 10 mg/kg diet	0.2 mg/kg + 15 mg/kg diet
Soybean meal^a^	35.5	35.5	35.5	35.5
Fish meal^a^	20.0	20.0	20.0	20.0
Groundnut meal^a^	10.0	10.0	10.0	10.0
Wheat flour^a^	23.47	23.4698	23.4698	23.4698
Cod liver oil^a^	6.0	5.8	5.6	5.4
CMC^b^	2.0	2.0	2.0	2.0
Vitamin and mineral mix*	2.0	2.0	2.0	2.0
Lecithin	1.0	1.0	1.0	1.0
Vitamin C^d^	0.03	0.03	0.03	0.03
Selenium Nanoparticles (Se-NPs)	0	0.0002	0.0002	0.0002
EPA + DHA	0	0.2	0.4	0.6
**Proximate analysis of experimental feed**				
CP^1^	36.49 ± 0.69	35.54 ± 0.580.46	35.64 ±	34.96 ± 0.11
EE^2^	9.27 ± 0.30	11.41 ± 0.55	11.45 ± 0.11	11.89 ± 0.63
Ash	8.48 ± 0.15	10.19 ± 0.30	10.74 ± 0.14	10.92 ± 0.47
TC^3^	45.76 ± 0.35	42.86 ± 0.90	42.17 ± 0.33	42.23 ± 0.38
OM^4^	91.52 ± 0.15	89.81 ± 0.30	89.26 ± 0.14	89.08 ± 0.47
DM^5^	92.93 ± 0.61	92.36 ± 0.30	92.74 ± 0.04	91.83 ± 0.13
DE^6^	393.90 ± 0.58	393.48 ± 1.98	391.40 ± 0.70	391.99 ± 3.53

CP^1^—crude protein; EE^2^—ether extract; TC^3^—total carbohydrate; OM^4^—organic matter, DM^5^—dry matter, DE^6^—digestible energy.

Digestible energy (DE) (Kcal/100 g) = (% CP × 4) + (% EE × 9) + (TC × 4).

Data expressed as mean ± SE, n = 3.

*Manual prepared selenium free mineral mixture; Composition of vitamin mineral mix (quantity/250 g starch powder): vitamin A 550,000 IU; vitamin D3 110,000 IU; vitamin B1:20 mg; vitamin E 75 mg; vitamin K 100 mg; vitamin B12 0.6 mcg; calcium pantothenate 250 mg; nicotinamide 1000 mg; pyridoxine: 100 mg; Mn 2700 mg; I 100 mg; Zn: 500 mg; Fe 750 mg; Cu 200 mg; Co 45 mg; Ca 50 g; P 30 g.

^a^Procured from local market, ^b^Himedia Ltd, ^c^*Prepared manually and all components from Himedia Ltd, ^c^SD Fine Chemicals Ltd., India.

### Experimental procedure

*Pangasianodon hypophthalmus* (weight, 4.73 ± 0.52 g, length, 4.11 cm) were procured from West Bengal, India. Fish was transported in healthy condition to Central Wet Laboratory facility of ICAR-NIASM, Baramati, Pune. Before stocking in fibre-reinforced plastic tanks, fishes were treated with salt (1%) and KMnO_4_ solution (2 ppm) and then acclimatized for 40 days before the commencement of the experiment. The fishes were fed with basal diet until the commencement of the experiment. Water quality were also recorded periodically using the methods suggested by the American Public Health Association, APHA^45^. The experiment was conducted in triplicates in twenty-four (24) tanks (capacity 150 L) with eighteen (18) fish (mean weight of 5.6 ± 1.68) in each replicate for 105 days. Eight (8) treatments were designed as follows and shown in Table 2.

**Table 2:** Details of the experimental design.

Treatment number (s)	Treatments	Treatments details	Symbols
1	Control group	No exposure to stressors (arsenic + temperature and no supplementation of Se-NPs and EPA + DHA) and fed with control diet	Control
2	Arsenic (2.68 mg L^−1^) and high temperature (34 °C) exposure	Concurrent exposure to arsenic (2.68 mg L^−1^) and high temperature (34 °C) and fed with control diet	As + T
3	Se-NPs at 0.2 mg kg^−1^ diet with EPA + DHA at 0.2%	Fed with Se-NPs at 0.2 mg kg^−1^ and mixture of EPA + DHA at 0.2%	Se-NPs + ED-0.2%
4	Se-NPs at 0.2 mg kg^−1^ diet with EPA + DHA at 0.4%	Fed with Se-NPs at 0.2 mg kg^−1^ and mixture of EPA + DHA at 0.4%	Se-NPs + ED-0.4%
5	Se-NPs at 0.2 mg kg^−1^ diet with EPA + DHA at 0.6%	Fed with Se-NPs at 0.2 mg kg^−1^ and mixture of EPA + DHA at 0.6%	Se-NPs + ED-0.6%
6	Fed with Se-NPs at 0.2 mg kg^−1^ diet with EPA + DHA at 0.2% and concurrent exposure to arsenic and high temperature	Fed with Se-NPs at 0.2 mg kg^−1^ and mixture of EPA + DHA at 0.2% and treated under concurrent exposure to arsenic 2.68 mg L^−1^) and high temperature (34 °C)	Se-NPs + ED-0.2%
7	Fed with Se-NPs at 0.2 mg kg^−1^ diet with EPA + DHA at 0.4% and concurrent exposure to arsenic and high temperature	Fed with Se-NPs at 0.2 mg kg^−1^ and mixture of EPA + DHA at 0.4% and treated under concurrent exposure to arsenic 2.68 mg L^−1^) and high temperature (34 °C)	Se-NPs + ED-0.4%
8	Fed with Se-NPs at 0.2 mg kg^−1^ diet with EPA + DHA at 0.6% and concurrent exposure to arsenic and high temperature	Fed with Se-NPs at 0.2 mg kg^−1^ and mixture of EPA + DHA at 0.6% and treated under concurrent exposure to arsenic 2.68 mg L^−1^) and high temperature (34 °C)	Se-NPs + ED-0.6%

Two-thirds of the tank’s water was manually exchanged on every alternate day, the arsenic concentration was maintained, and continuous aeration was provided with a compressed air pump. Feed was presented to fish in each tank until they reached apparent satiation, when feeding activity ceased. The amount of feed consumed by the fish and uneaten feed as well as faecal matters in each tank was removed daily by siphoning. In the stressors group arsenic was added as 1/10th of LC_50_ 2.68 mg L^−1^ of arsenic^3^ and temperature (34 °C) was maintained with thermostatic heater^3^.

### Green synthesis and characterization of Se-NPs

**Figure 1:**
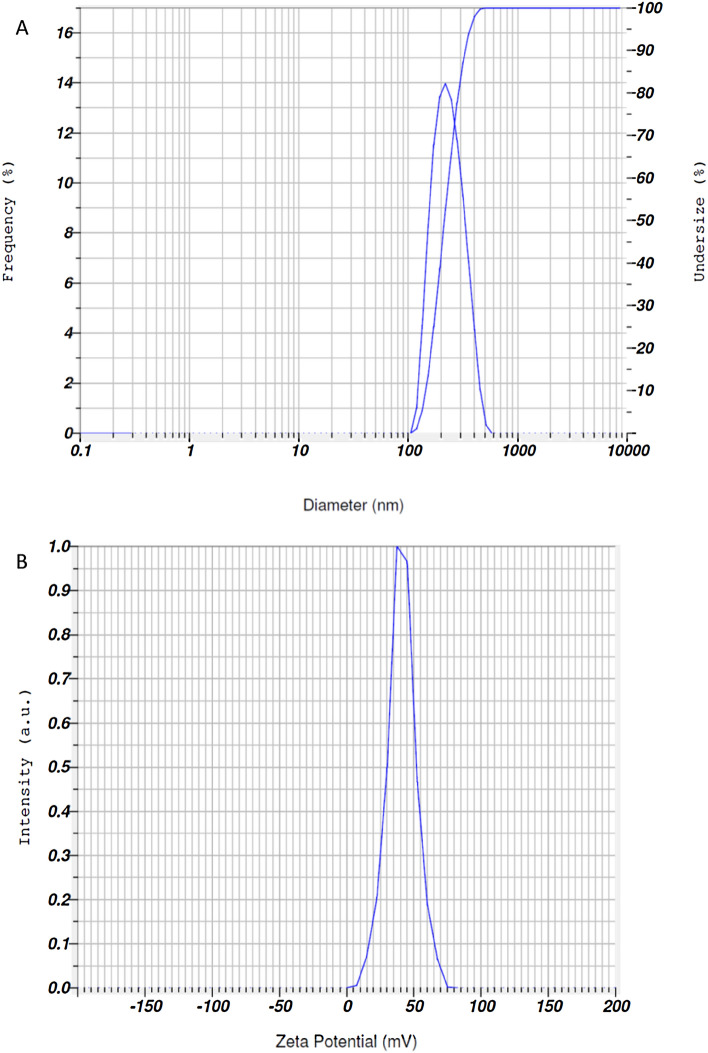
(**A**) Size of selenium-nanoparticles (203.5 nm) and (**B**) zeta potential − 41.8 mV.

Fish gill tissue was used for the synthesis of Se-NPs. The tissues were neatly dissected out and cleaned carefully under running water, cut into several small pieces and homogenized. Tissues homogenates were centrifuged at 5000–6000 rpm for 15 min at 4 °C. The supernatant was obtained and filtered. After that, supernatant was mixed with 2 M sodium selenite (200 ml) on a shaker for 96 h. The final solution was centrifuged at 6000 rpm at 15 min at 4 °C and then pellet was obtained. The pellet was isolated and dried for 5 h. Before diet preparation, Se-NPs were crushed in to a fine powder^35,36^. Se-NPs were characterized through a spectrophotometer in an absorption spectrum at 360–380 nm. Particles size of 203.5 nm and zeta potential − 41.8 mV (Fig. 1A,B) was confirmed through Nanoparticles Analyzer (Horiba Scientific Nanoparticles Analyzer, nano-partica SZ-100 series Kyoto, Japan) at 25 °C.

### Tissue homogenate preparation and blood collection

Fishes were anesthetized with clove oil (100 µl L^−1^) before collecting the gill, kidney, liver and brain tissues. The collected tissues were homogenized in chilled sucrose (5% w/v, 0.25 M) and EDTA solution (1 mM) using tissue homogenizer (Omni Tissue Master Homogenize, Kennesaw, GA). The sample containing tube was kept on ice during homogenization to avoid denaturation of the enzyme activities. The sample homogenates were centrifuged for supernatant collection at 5000×*g* for 15 min at 4 °C in a cooling centrifuge (Eppendorf AG, 5430R, Hamburg, Germany). After that, the supernatant were stored at − 20 °C until further analysis. Blood was also collected from four (4) fish and used for serum preparation (heparin free syringe) and blood from three (3) fish was collected with heparinized syringe to avoid blood clotting. The tissue protein was also determined using Lowry method^46^.

### Sample preparation for analysis of arsenic and selenium

Muscle, liver, gill, kidney and brain were collected for determination of arsenic concentration. Elements such as selenium (Se), lithium (Li), sodium (Na), magnesium (Mg), potassium (K), calcium (Ca), vanadium (V), chromium (Cr), manganese (Mn), iron (Fe), cobalt (Co), nickel (Ni), copper (Cu), zinc (Zn), gallium (Ga), arsenic (As), rubidium and molybdenum (Mo) were analyzed in experimental diet. The tissues and diets were processed in microwave digestion system (Microwave Reaction System, Multiwave PRO, Anton Paar GmbH, Austria, Europe) using HNO_3_ and H_2_O_2_ in 5:1 ratio. After acidic digestion the digested sample were filtered using 0.45 µm pore size filter paper and made up to volume of 50 mL for final process. Water used for experiments was acidified with HNO_3_ (100 µL HNO_3_ in 10 mL water samples) and used for arsenic determination. Then samples were analyzed in an Inductively Coupled Plasma Mass Spectrometry (ICP-MS) (Agilent 7700 series, Agilent Technologies, USA). The multi-element standard was used for calibration curve at R^2^ > 0.999^47,48^.

### Growth performance

Weight gain (%), feed conversion ratio (FCR), specific growth rate, protein efficiency ratio (PER), daily growth index (DGI), thermal growth coefficient (TGC) and relative feed intake (RFI) were determined in the study. The weight of the fish was observed on every 15 days intervals up to 105 days.

$$\begin{aligned} {\text{Weight}}\,{\text{gain}}\,\left( \% \right) & = {\text{Final}}\,{\text{body}}\,{\text{weight}}\,\left( {{\text{FBW}}} \right) - {\text{Initial}}\,{\text{body}}\,{\text{weight}}\,\left( {{\text{IBW}}} \right)/{\text{Initial}}\,{\text{body}}\,{\text{weight}}\,\left( {{\text{IBW}}} \right) \times {1}00 \\ {\text{FCR}} & = {\text{Total}}\,{\text{dry}}\,{\text{feed}}\,{\text{intake}}\,\left( {\text{g}} \right)/{\text{Wet}}\,{\text{weight}}\,{\text{gain}}\,\left( {\text{g}} \right) \\ {\text{SGR}} & = {1}00\,\left( {{\text{ln}}\,{\text{FBW}} - {\text{ln}}\,{\text{IBW}}} \right)/{\text{number}}\,{\text{of}}\,{\text{days}} \\ {\text{PER}} & = {\text{Total}}\,{\text{wet}}\,{\text{weight}}\,{\text{gain}}\,\left( {\text{g}} \right)/{\text{crude}}\,{\text{protein}}\,{\text{intake}}\,\left( {\text{g}} \right) \\ {\text{Relative}}\,{\text{feed}}\,{\text{intake,}}\,\left( {{\text{FI}}} \right)\,\left( {\% /{\text{d}}} \right) & = {1}00 \times \left( {{\text{TFI}}/{\rm I}{\text{BW}}} \right) \\ {\text{Daily}}\,{\text{growth}}\,{\text{index}},\,{\text{DGI}}\,\left( \% \right) & = \left( {{\text{FBW}}^{{{1}/{3}}} {-}{\text{IBW}}^{{{1}/{3}}} } \right)/{\text{days}} \times {1}00 \\ {\text{Thermal}}\,{\text{growth}}\,{\text{coefficient}},\,\left( {{\text{TGC}}} \right) & = \left( {{\text{FBW}}^{{{1}/{3}}} {-}{\text{IBW}}^{{{1}/{3}}} } \right) \times \left( {\Sigma {\text{D}}0} \right)^{{ - {1}}} \end{aligned}$$

where $$\Sigma {\text{D}}0\,{\text{is}}\,{\text{the}}\,{\text{thermal}}\,{\text{sum}}\,({\text{feeding}}\,{\text{days}} \times {\text{average}}\,{\text{temperature}},^\circ {\text{C}})$$

### Antioxidant enzyme activities

Activity of superoxide dismutase (EC 1.15.1.1) was estimated using method described by Misra and Fridovich^49^. Activity of catalase (EC 1.11.1.6) was determined using method described by Takahara et al.^50^. Glutathione-s-transferase, GST (EC 2.5.1.18) and glutathione peroxidase, GPx (EC 1.11.1.9) were determined using method described by Habing et al.^51^, and Paglia and Valentine^52^, respectively.

### Lipid peroxidation (LPO)

LPO was determined using the method of Uchiyama and Mihara^53^. Briefly, 0.25 mL of liver and kidney tissues homogenates were mixed with 25 μL of 10 mM butylated hydroxytoluene (BHT) to which 3 mL phosphoric acid (1%) with 1 mL of 0.67% thiobarbituric acid (TBA) were added. The homogenate was then incubated at 90 °C for 45 min and absorption was read at 535 nm in spectrophotometer.

### Neurotransmitter enzyme activity

Acetylcholine esterase (AChE) (EC. 3.1.1.7) activity was measured using the Hestrin et al.^54^ and modified by Augustinsson^55^.

### Cortisol and HSP-70

Serum cortisol and HSP 70 were determined using ELISA kit (Cortisol EIA kit, catalogue no. 500360, Cayman Chemicals, USA; HSP 70 kit EKS-700B, Bioguenix/Enzo Life Science, Mumbai, India). The assay was performed as per instruction provided with the kit.

### Ascorbic acid (vitamin C)

Ascorbic acid was estimated from brain and muscle tissue, followed by the method of Roe and Keuther^56^.

### Nitroblue tetrazolium (NBT), serum protein and A:G ratio

NBT activities were determined as followed as Secombes^57^ and modified by Stasiack and Baumann^58^. The serum protein was estimated by using a protein estimation kit. Albumin was estimated by method of Doumas et al.^59^ and globulin was quantified by subtracting albumin values from total plasma protein.

### Myeloperoxidase content (MPO) and total immunoglobulin level

The myeloperoxidase was quantified as method of Quade and Roth^60^ with some modifications^61^ and total immunoglobulin level was determined using method of Anderson and Siwicki^62^.

### Blood glucose

The determination of blood glucose was determined as per the method of Nelson^63^ and Somoyogi^64^. The final reading was obtained at 540 nm against the blank.

### Lipid profiling

Total lipid was determination as per method of Bligh and Dyer^65^. Similarly, phospholipid was determined as per method of Bartlett^66^ and modified by Marinetti^67^. Total cholesterol was determined as per the procedure of Henly^68^. Further, triglyceride was calculated based on total lipid, phospholipid and cholesterol. The very low density lipoprotein (VLDL) was calculated by dividing triglyceride by 5.

### Challenge study with *Aeromonas hydrophila*

*P. hypophthalmus* was infected with *Aeromonas hydrophilla* (Lot no. 637-51-5 and Ref 0637P, HiMedia, Mumbai) after 105 days experimental trial. *A. hydrophila* was culture in nutrient broth for 24 h at 37 °C in an incubator and the culture was harvested and after centrifuging the culture broth at 6000 rpm for 15 min at 4 °C. The harvested cells were washed thrice in PBS (pH 7.2) and finally diluted to obtain 10^8^ CFU mL^−1^. Thereafter, 0.15 mL of bacterial suspension was injected to fishes and observed morality up to 7 days. The tissues were collected from morbid fish for confirmation of *A. hydrophilla*. Cumulative mortality and relative survival were obtained as follows:

$$\begin{aligned} {\text{Cumulative}}\,{\text{mortality }}\left( \% \right) & = \frac{{{\text{Total}}\,{\text{mortality}}\,{\text{in}}\,{\text{each}}\,{\text{treatment}}\,{\text{after}}\,{\text{challenge}}}}{{{\text{Total}}\,{\text{no}}.\,{\text{of}}\,{\text{fish}}\,{\text{challenged}}\,{\text{for}}\,{\text{the}}\,{\text{same}}\,{\text{treatments}}}} \times {1}00 \\ {\text{Relative}}\,\% \,{\text{survival}} & = \frac{{{\text{Mortality}}\,\left( \% \right)\,{\text{Control}}{-}{\text{Mortality}}\,\left( \% \right)\,{\text{Treatment}}}}{{{\text{Mortality}}\,\left( \% \right){\text{Control}}}} \times {1}00 \\ \end{aligned}$$

### Statistical analysis

Data were analyzed using Statistical Package for Social Sciences program version 16.0 (SPSS Inc., Chicago, IL, USA). The data were expressed as mean ± standard error of mean and tested for normality and homogeneity of variance using the Shapiro–Wilk’s and Levene’s test, respectively. When both tests were satisfied, one-way ANOVA (Analysis of variance) with Duncan’s multiple range tests (DMRT) was employed to test the statistically significant difference at p < 0.05.

## Results

### Concurrent exposure to arsenic and temperature elicits primary stress response (cortisol) but alleviate by dietary EPA + DHA and Se-NPs in *P. hypophthalmus*

**Figure 2:**
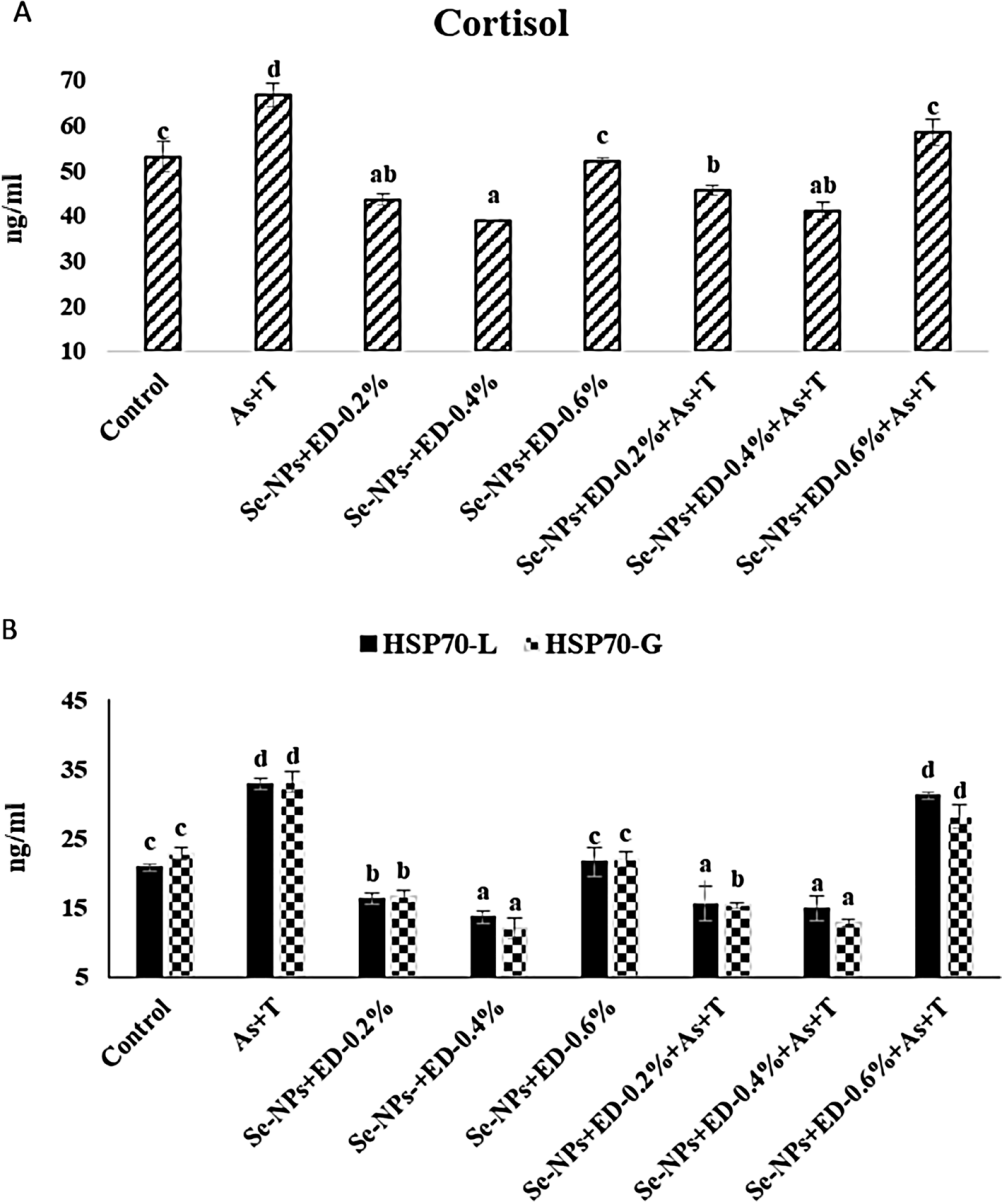
(**A**, **B**) Mitigation of primary and secodary stress response (Cortisol and HSP 70) through dietary supplementation of selenium nanoparticles, eicosapentanoic acid and dicosahexanoic acid fed to *P. hypophthalmus* reared in control or under arsenic and high temperature (As + T) for 105 days. Within endpoints and groups, bars with different superscripts differ significantly (a–d) cortisol (p = 0.032), HSP-L (p = 0.026), HSP-G (p = 0.014). Data expressed as Mean ± SE (n = 3).

The results of cortisol are shown in the Fig. 2A. Primary stress response is the resultant of first indicator of stress induced by arsenic and high temperature in fish. In the present investigation the cortisol was noticeably elevated (p = 0.0032) with concurrent exposure to arsenic (As) and high temperature (34 °C) (As + T) when not fed with supplemented diet, in comparison to the control group. Cortisol was significantly reduced (p < 0.01) with supplementation of Se-NPs (0.2 mg kg^−1^ diet) and EPA + DHA at 0.2 and 0.4% with or without exposure to As + T in comparison to control group. However, group supplemented with Se-NPs at 0.2 mg kg^−1^ diet and EPA + DHA at 0.6% with or without stressors (As + T) was similar to control group.

### Concurrent exposure to arsenic and temperature elicits secondary stress response (HSP 70; Oxidative stress; LPO) but alleviate by dietary EPA + DHA and Se-NPs in *P. hypophthalmus*

Chaperon protein are highly conservative in nature. In the present investigation, we have studied heat shock protein 70 (HSP 70) in liver and gill tissues (Fig. 2B). The HSP 70 in liver (p = 0.026) and gill (p = 0.014) were noticeably elevated in the group concurrently exposed to As + T and fed with control diet in comparison to unexposed group (control group). Se-NP at 0.2 mg kg^−1^ and EPA + DHA at 0.4% diet significantly reduced the HSP 70 in liver and gill tissues with or without exposure to As + T in comparison to unexposed group (control group) and group exposed to As + T and fed with control diet. Gills and livers of the fish exposed to As and thermal stress and fed with diet containing 0.6% EPA + DHA showed highest HSP-70, which was also statistically similar to the fish exposed to As and thermal stress alone.

CAT activity in liver (Table 3) was significantly higher (p = 0.0023) in fish concurrently exposed to As + T and fed with control diet in comparison to control and other supplemented group except Se-NPs at 2 mg kg^−1^ and EPA + DHA at 0.6% diet and exposed to As + T. Whereas, CAT activity was noticeably lower (p < 0.01) in supplemented group fed with Se-NPs at 0.2 mg kg^−1^ and EPA + DHA at 0.4% in comparison to all other groups. Whereas, in kidney tissue, the remarkably highest (p = 0.002) activities was observed in control fed and concurrently exposed to As + T followed by group fed with Se-NPs at 0.2 mg kg^−1^ with EPA + DHA at 0.6% diet with or without exposure to As + T. The CAT activities were significantly (p < 0.01) reduced with supplementation of Se-NPs and EPA + DHA at 0.4% diet in comparison to all other groups. Similarly, in case of gill catalase, highest activity was observed (p = 0.014) in group exposed to As + T and fed with control diet in comparison to unexposed group. The supplementation of Se-NPs at 0.2 mg kg^−1^ with EPA + DHA at 0.4% significantly reduced the catalase activity in gill tissues with or without As + T exposure, followed by 0.2% fed group in comparison to control group, stressors group and Se-NPs and EPA + DHA at 0.6%. Similarly, superoxide dismutase (SOD) activities in liver (p = 0.034) and gill (p = 0.027) were significantly elevated in group concurrently exposed to As + T and fed with control diet group. Further, the activity of SOD in liver was noticeably reduced (p < 0.01) with supplementation of Se-NPs and EPA + DHA at 0.4 and 0.2% diet group in comparison to all other treatments. In case of kidney, the highest activities were observed in the group concurrently exposed to As + T and fed with control diet as well as Se-NPs and EPA + DHA at 0.6% diet group. Whereas, the activities were significantly reduced (p = 0.043) with application of Se-NPs and EPA + DHA at 0.4% and 0.2% diet in comparison to stressors group. The activity of SOD in kidney was similar in control group and supplemented group (Se-NPs and EPA + DH at 0.4 and 0.2%). Whereas, in case of gill, the supplementation with Se-NPs and EPA + DHA at 0.4, 0.2 and 0.6% diet group was did not affect SOD activity in comparison to control group (Table 3). GST activities in gill (p = 0.041) and kidney (p = 0.0013) were significantly higher in group concurrently exposed to As + T and fed with control diet as well as Se-NPs and EPA + DHA at 0.6% in comparison to control group. Whereas, in case of liver (p = 0.011) the highest activities were observed in the group concurrently exposed to As + T and fed with control diet and Se-NPs at 0.2 mg kg^−1^ and EPA + DHA at 0.6% diet group then unexposed group. Dietary supplementation with Se-NPs at 0.2 mg kg^−1^ and EPA + DHA at 0.4 and 0.2% with or without stressors (As + T) significantly reduced (p < 0.01) the GST activities in liver and kidney, in comparison to all other treatments group. Similarly, gill GST activities were remarkably reduced (p < 0.01) with supplemented group of Se-NPs and EPA + DHA at 0.4% diet group in comparison to other treatments (Table 4). Glutathione peroxidase (GPx) activities in liver, gill and kidney were significantly enhanced (p < 0.01) with exposure to As + T and fed with control diet and Se-NPs and EPA + DHA at 0.6% diet group in comparison to unexposed group (control group). GPx in liver (p = 0.0035), gill (p = 0.041) and kidney (p = 0.0023) were noticeably reduced (p < 0.01) with supplementation of Se-NPs at 0.2 mg kg^−1^ and EPA + DHA at 0.4% and 0.2% diet group with or without stressors. The supplemented group with Se-NPs at 0.2 mg kg^−1^ and EPA + DHA at 0.6% diet, without exposure to stressors, showed GPx activity similar to control diet group in liver, gill and kidney tissues, whereas, with exposure to stressors (As + T) the group (Se-NPs at 0.2 mg kg^−1^ diet and EPA + DHA at 0.6%) showed a significantly higher GPx activity in comparison to control, but similar to that of the fish exposed to As + T and fed with control diet (Table 4).

**Table 3:** Mitigation of secondary stress response (catalase and superoxide dismutase) through dietary supplementation of selenium nanoparticles, eicosapentanoic acid and dicosahexanoic acid fed to *P. hypophthalmus* reared in control or under arsenic and high temperature for 105 days.

Treatments	Non-stressors	Stressors (arsenic and temperature)	Non-stressors			Stressors (arsenic and temperature)			P-value
Diets	Control	Control	Se-NPs + EPA + DHA-0.2%	Se-NPs + EPA + DHA-0.4%	Se-NPs + EPA + DHA-0.6%	Se-NPs + EPA + DHA-0.2%	Se-NPs + EPA + DHA-0.4%	Se-NPs + EPA + DHA-0.6%	
CAT-Liver	3.96^d^ ± 0.26	6.70f. ± 0.61	2.29^c^ ± 0.19	0.91^a^ ± 0.13	4.24^e^ ± 0.31	2.13^c^ ± 0.24	1.29^b^ ± 0.11	6.39f. ± 0.18	0.0023
CAT-Gill	3.11^c^ ± 0.40	8.44^d^ ± 0.61	1.61^b^ ± 0.25	0.78^a^ ± 0.08	3.04c ± 0.53	1.52b ± 0.28	0.81a ± 0.07	3.21^c^ ± 0.59	0.014
CAT-Kidney	5.73^c^ ± 0.76	9.01^d^ ± 0.38	2.68^b^ ± 0.29	1.04^a^ ± 0.15	5.09^c^ ± 0.41	2.22^b^ ± 0.31	1.20^a^ ± 0.19	9.47^d^ ± 0.92	0.002
SOD-Liver	66.0^b^ ± 1.82	75.04^d^ ± 1.14	66.71^b^ ± 1.16	62.02^a^ ± 1.13	70.85^c^ ± 1.09	66.75^b^ ± 1.50	62.59^a^ ± 0.58	72.07^c^ ± 0.91	0.034
SOD-Gill	31.49^a^ ± 1.23	38.71^b^ ± 0.58	30.63^a^ ± 1.44	32.01^a^ ± 1.40	30.31^a^ ± 1.84	32.26^a^ ± 0.80	30.15^a^ ± 1.96	30.84^a^ ± 1.46	0.027
SOD-Kidney	41.16^a^ ± 1.29	46.66^b^ ± 2.16	42.32^a^ ± 1.54	40.57^a^ ± 0.98	43.34^ab^ ± 1.25	40.97^a^ ± 0.95	41.0^a^ ± 1.52	47.15^b^ ± 1.46	0.043

Values in the same row with different superscript (a, b, c, d, e, f) differ significantly. Data expressed as Mean ± SE (n = 6). Catalase and SOD: Units/mg protein.

**Table 4:** Mitigation of secondary stress response (glutathione-s-transferase (GST), glutathione peroxidase (GPx) and lipid peroxidation (LPO)) through dietary supplementation of selenium nanoparticles, eicosapentanoic acid and dicosahexanoic acid fed to *P. hypophthalmus* reared in control or under arsenic and high temperature for 105 days.

Treatments	Non-stressors	Stressors (arsenic and temperature)	Non-stressors			Stressors (arsenic and temperature)			P-value
Diets	Control	Control	Se-NPs + EPA + DHA-0.2%	Se-NPs + EPA + DHA-0.4%	Se-NPs + EPA + DHA-0.6%	Se-NPs + EPA + DHA-0.2%	Se-NPs + EPA + DHA-0.4%	Se-NPs + EPA + DHA-0.6%	
GST-Liver	0.29^b^ ± 0.01	0.44^d^ ± 0.02	0.19^a^ ± 0.02	0.14^a^ ± 0.01	0.27^b^ ± 0.03	0.18^a^ ± 0.01	0.12^a^ ± 0.03	0.36^c^ ± 0.01	0.011
GST-Gill	0.35^b^ ± 0.04	0.46^c^ ± 0.02	0.24^ab^ ± 0.02	0.14^a^ ± 0.01	0.33^b^ ± 0.06	0.23^ab^ ± 0.01	0.17^a^ ± 0.01	0.43^c^ ± 0.05	0.041
GST-Kidney	0.25^c^ ± 0.01	0.45^d^ ± 0.03	0.19^b^ ± 0.02	0.13^a^ ± 0.01	0.23^c^ ± 0.02	0.18^b^ ± 0.03	0.11^a^ ± 0.01	0.41^d^ ± 0.04	0.0013
GPx-Liver	4.90^c^ ± 0.57	9.19^e^ ± 0.51	2.19^b^ ± 0.25	1.52^a^ ± 0.17	4.25^c^ ± 0.28	2.09^b^ ± 0.40	1.55^a^ ± 0.21	6.00^d^ ± 0.46	0.0035
GPx-Gill	3.98^c^ ± 0.24	8.55^e^ ± 0.31	2.94^b^ ± 0.49	1.46^a^ ± 0.14	3.67^c^ ± 0.35	2.97^b^ ± 0.28	1.57^a^ ± 0.16	7.43^d^ ± 0.75	0.041
GPx-Kidney	8.49^c^ ± 0.47	12.25^d^ ± 1.27	3.33^b^ ± 0.45	1.54^a^ ± 0.26	7.90^c^ ± 0.58	3.33^b^ ± 0.34	1.56^a^ ± 0.25	11.12^d^ ± 0.61	0.0023
LPO-Liver	17.94^c^ ± 0.56	39.61^e^ ± 1.67	12.59^b^ ± 0.49	10.66^a^ ± 0.65	18.59^c^ ± 0.71	18.11^c^ ± 1.12	9.77^a^ ± 0.42	30.38^d^ ± 1.78	0.028
LPO-Gill	11.38^b^ ± 1.44	17.47^c^ ± 0.41	8.26^a^ ± 0.65	7.08^a^ ± 0.43	10.19^b^ ± 0.44	7.46^a^ ± 0.32	7.90^a^ ± 0.43	17.57^c^ ± 0.,57	0.023
LPO-Kidney	23.72^c^ ± 1.05	32.95^d^ ± 0.97	16.85^b^ ± 0.38	10.28^a^ ± 0.70	21.39^c^ ± 0.51	15.48^b^ ± 1.13	10.33^a^ ± 0.70	31.07^d^ ± 2.16	0.034

Values in the same row with different superscript (a, b, c, d, e) differ significantly. Data expressed as Mean ± SE (n = 6). GST and GPx: Units/mg protein. LPO: n mole TBARS formed/h/mg protein.

Lipid peroxidation (LPO) level in gill (p = 0.023), liver (0.028) and kidney (p = 0.034) tissues were noticeably higher (p < 0.01) in group exposed to As + T and fed with control diet as well as Se-NPs at 0.2 mg kg^−1^ and EPA + DHA at 0.6% diet in comparison to control group. The LPO in liver and kidney were significant reduced (p < 0.01) with dietary supplementation of Se-NPs and EPA + DHA at 0.4% diet group in comparison to all other treatment groups. Whereas, LPO in gill was significantly lowered (p = 0.023) with dietary supplementation of Se-NPs and EPA + DHA at 0.2 or 0.4%, with or without stressors, (Table 4) in comparison to all other treatment groups.

### Concurrent exposure to arsenic and temperature elicits secondary stress response (Acetylcholine esterase, Vitamin C) but alleviate by dietary EPA + DHA and Se-NPs in *P. hypophthalmus*

**Figure 3:**
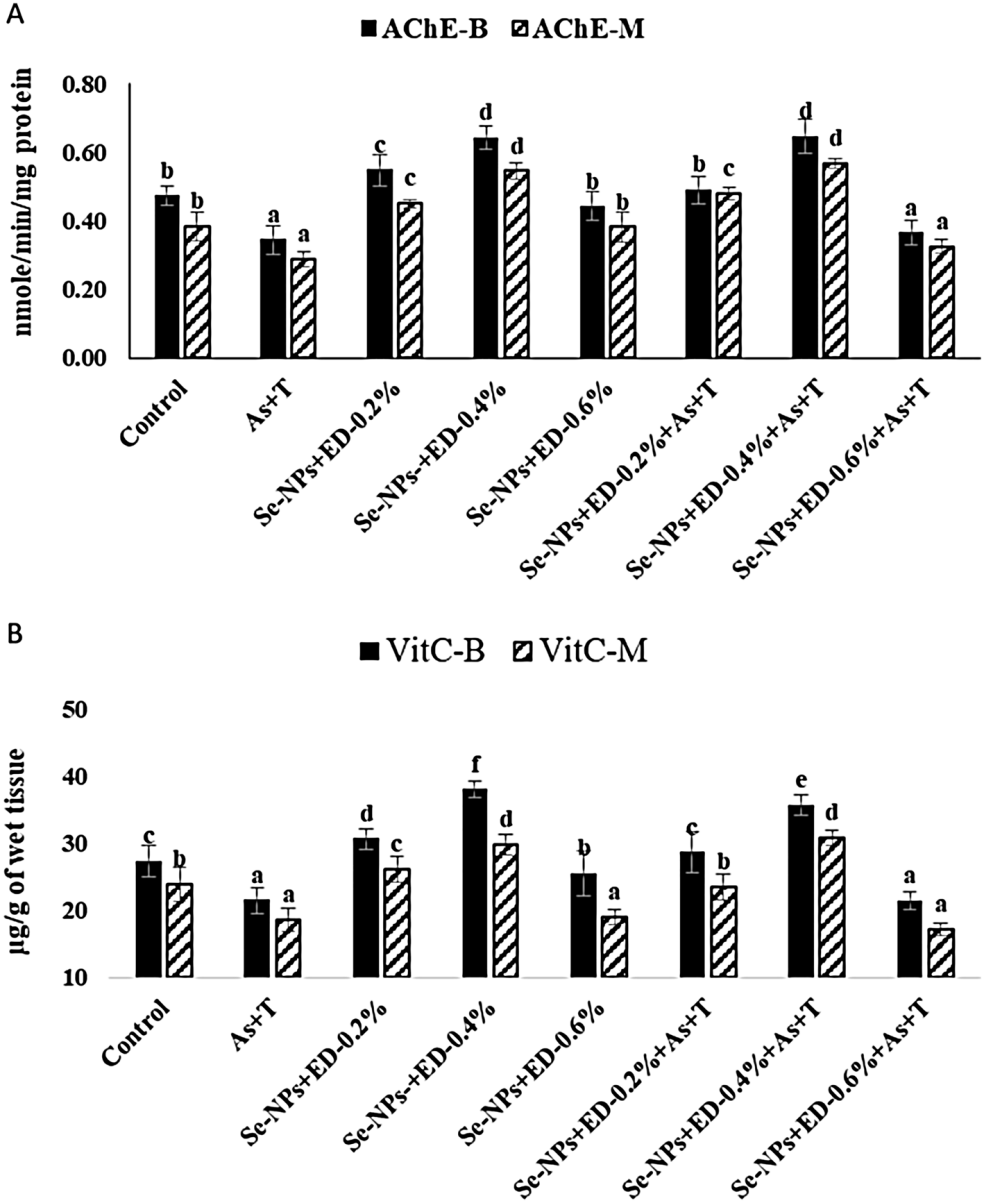
(**A**, **B**) Mitigation of secondary stress response (acetylcholine esterase and vitamin C) through dietary selenium nanoparticles, eicosapentanoic acid and dicosahexanoic acid fed to *P. hypophthalmus* reared in control or under arsenic and high temperature (As + T) for 105 days. Within endpoints and groups, bars with different superscripts differ significantly (a–f) AChE-B (p = 0.0028), AChE-M (p = 0.029), VitC-B (p = 0.0056), VitC-M-G (p = 0.038). Data expressed as Mean ± SE (n = 6).

Figure 3A presents the AChE activity in brain and muscle tissues of *P. hypophthalmus* fed with EPA + DHA and Se-NPs reared in control or under As + T. AChE activities in brain (p = 0.0028) and muscle (p = 0.029) tissues were noticeably inhibited (p < 0.01) with concurrent exposure to As + T and fed with control diet or Se-NPs with EPA + DHA at 0.6%. Whereas AChE activity in muscle and brain was noticeably enhanced (p < 0.01) with supplementation of Se-NPs and EPA + DHA at 0.4% diet group with or without exposure to As + T in comparison to other treatments. Similarly, Vitamin C in muscle (p = 0.038) and brain (p = 0.0056) was significantly reduced with exposure to As + T and fed with control diet as well as Se-NPs and EPA + DHA at 0.6% diet group in comparison to unexposed group (control group). Further, Vitamin C in brain and muscle was remarkably enhanced (p < 0.01) with dietary supplementation of Se-NPs and EPA + DHA at 0.4% diet group with or without exposure to stressors (As + T), in comparison to other treatments. Results also revealed that supplementation of Se-NPs at 0.2 mg kg^−1^ and EPA + DHA at 0.6% diet group noticeably reduced the Vit C in brain and muscle tissues with or without exposure to As + T in comparison to other supplemented and control diet group (Fig. 3B).

### Concurrent exposure to arsenic and temperature elicits secondary stress response (total protein, albumin, globulin, A:G ratio, NBT, blood glucose, total immunoglobulin and myeloperoxidase) but alleviate by dietary EPA + DHA and Se-NPs in *P. hypophthalmus*

Table 5 summarizes the results of immunological attributes of *P. hypophthalmus* exposed to As + T and fed with normal and experimental diets. Total protein was significantly enhanced (p = 0.001) with supplementation of Se-NPs at 0.2 mg kg^−1^ and EPA + DHA at 0.4% diet group with or without exposure to stressors (As + T), in comparison to other treatments including EPA + DHA at 0.2 and 0.6% diet group. Supplemented group with Se-NPs and EPA + DHA at 0.2% with or without exposure to As + T showed similar level of TP to control group. However, globulin (p = 0.0041) and NBT (p = 0.002) were significantly reduced with exposure to arsenic and high temperature (As + T) and fed with control diet or mixture of Se-NPs and EPA + DHA at 0.6%, in comparison to the other treatment groups. Moreover, the globulin and NBT were significantly higher in fish with supplementation of Se-NPs and EPA + DHA at 0.4% or 0.2%, with or without stressors (As + T), in comparison to control group, stressor group (As + T) and EPA + DHA at 0.6% diet group. Similarly, albumin and A: G ratio were significantly enhanced (p < 0.01) with exposure to As + T fed with control diet. Further, the albumin was significantly reduced (p = 0.038) with dietary supplementation of Se-NPs and EPA + DHA at 0.2, 0.4 and 0.6% diet group with or without exposure to As + T in comparison to unexposed group and stressor group and fed with control diet. Similarly, A:G ratio was significantly reduced with (p = 0.0051) supplementation of Se-NPs and EPA + DHA at 0.2 and 0.4% diet with or without exposure to As + T in comparison to control group and stressor group (As + T) (Table 4). Blood glucose of *P. hypophthalmus* exposed to As + T and fed with control diet was significantly elevated (p = 0.0063) in comparison to all other groups. Results showed that dietary supplementation of Se-NPs and EPA + DHA at 0.4 and 0.2% with or without exposure to stressors (As + T) were remarkably reduced the blood glucose level in comparison to control group and other supplemented group. The blood glucose in group fed with mixture of Se-NPs and EPA + DHA (without stressors) at 0.6% was similar to control diet group, whereas blood glucose was significantly higher in Se-NPs and EPA + DHA (with stressors) at 0.6% than control group (Table 5).

**Table 5:** Mitigation of secondary stress response (total protein, albumin, globulin, A:G ratio and blood glucose) through dietary supplementation of selenium nanoparticles, eicosapentanoic acid and dicosahexanoic acid fed to *P. hypophthalmus* reared in control or under arsenic and high temperature for 105 days.

Treatments	Non-stressors	Stressors (arsenic and temperature)	Non-stressors			Stressors (arsenic and temperature)			P-value
Diets	Control	Control	Se-NPs + EPA + DHA-0.2%	Se-NPs + EPA + DHA-0.4%	Se-NPs + EPA + DHA-0.6%	Se-NPs + EPA + DHA-0.2%	Se-NPs + EPA + DHA-0.4%	Se-NPs + EPA + DHA-0.6%	
Total Protein	0.52^b^ ± 0.02	0.40^a^ ± 0.01	0.56^b^ ± 0.03	0.63^c^ ± 0.06	0.35^a^ ± 0.03	0.54^b^ ± 0.01	0.65^c^ ± 0.03	0.35^a^ ± 0.01	0.001
Albumin	0.19^b^ ± 0.01	0.18^b^ ± 0.02	0.13^a^ ± 0.01	0.12^a^ ± 0.02	0.11^a^ ± 0.01	0.13^a^ ± 0.02	0.12^a^ ± 0.02	0.10^a^ ± 0.01	0.038
Globulin	0.33^b^ ± 0.03	0.22^a^ ± 0.01	0.43^c^ ± 0.03	0.51^d^ ± 0.06	0.24^a^ ± 0.02	0.42^c^ ± 0.05	0.53^d^ ± 0.03	0.24^a^ ± 0.02	0.0041
A:G Ratio	0.60^d^ ± 0.09	0.81^e^ ± 0.04	0.30^ab^ ± 0.01	0.25^a^ ± 0.05	0.45^c^ ± 0.11	0.34^b^ ± 0.04	0.24^a^ ± 0.01	0.43^c^ ± 0.03	0.0051
NBT	0.49^b^ ± 0.01	0.40^a^ ± 0.02	0.55^c^ ± 0.06	0.60^d^ ± 0.03	0.46^b^ ± 0.02	0.56^c^ ± 0.06	0.60^d^ ± 0.01	0.39^a^ ± 0.02	0.002
Blood glucose	128.81^c^ ± 10.01	158.99^e^ ± 2.45	121.56^b^ ± 1.75	105.46^a^ ± 3.14	127.36^c^ ± 5.15	121.84^b^ ± 1.50	105.14^a^ ± 7.92	148.93^d^ ± 8.08	0.0063

Values in the same row with different superscript (a, b, c, d, e) differ significantly. Total protein, albumin, globulin: g dL^−1^ Blood glucose: mgdL^−1^; Data expressed as Mean ± SE (n = 3).

**Figure 4:**
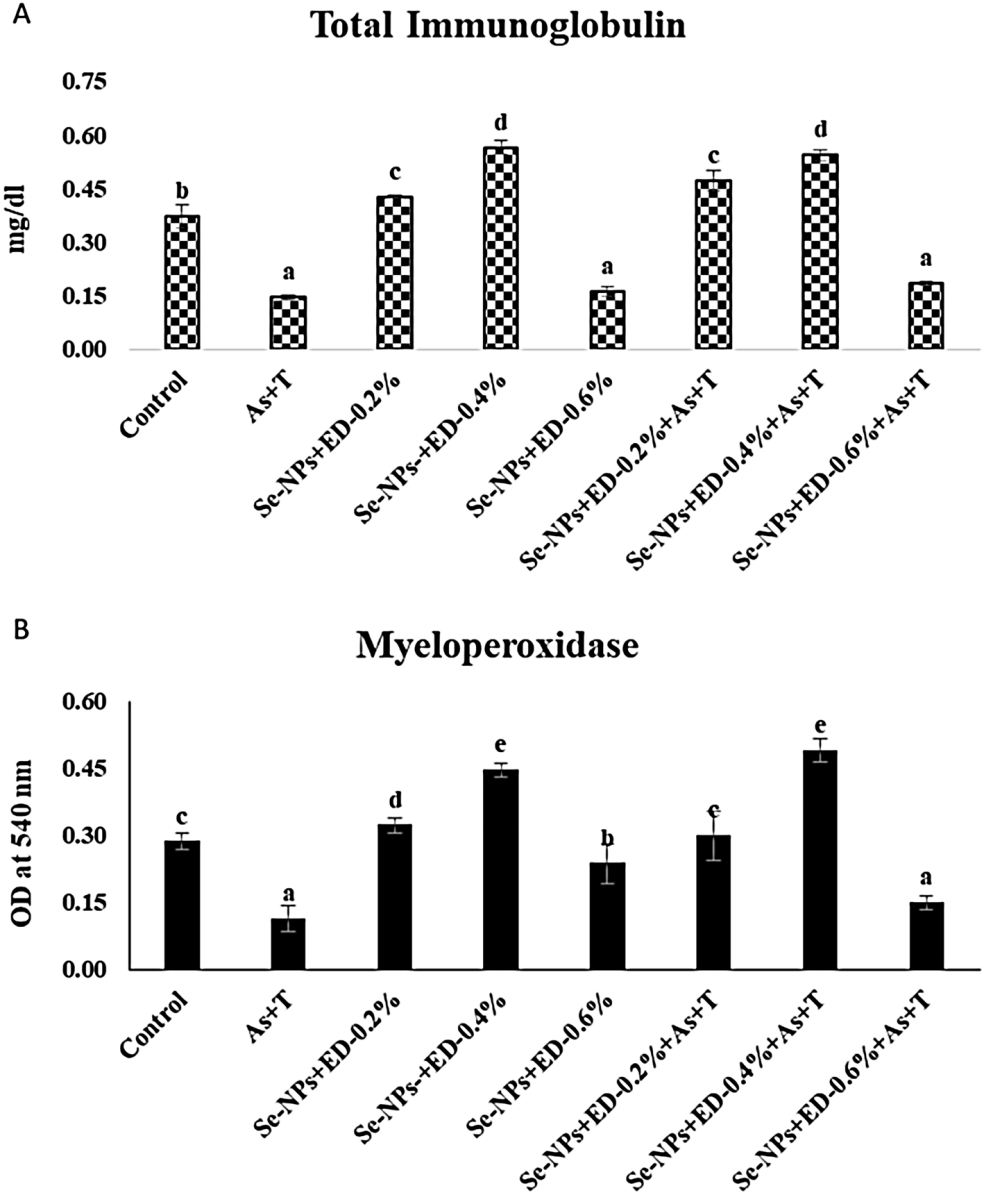
(**A**,**B**) Mitigation of secondary stress response (total immunoglobulin and myeloperoxiadase) through dietary supplementation of selenium nanoparticles, eicosapentanoic acid and dicosahexanoic acid fed to *P. hypophthalmus* reared in control or under arsenic and high temperature (As + T) for 105 days. Within endpoints and groups, bars with different superscripts differ significantly (a–f) Total immunoglobulin (p = 0.0042), Myeloperoxidase (p = 0.017). Data expressed as Mean ± SE (n = 6).

Other immunological attributes viz. total immunoglobulin and MPO are reported in the Fig. 4A, B. Total immunoglobulin (p = 0.0042) and MPO (p = 0.017) were significantly reduced with concurrent exposure to As + T and fed with control diet in comparison to control group and supplemented groups except EPA + DHA at 0.6 diet group in *P. hypophthalmus*. Whereas, total immunoglobulin was significantly enhanced (p = 0.0042) with supplementation of Se-NPs and EPA + DHA at 0.4 and 0.2% diet groups with or without exposure to stressors (As + T) in comparison to other treatments. Similarly, MPO was significantly enhanced (p = 0.017) with combinatorial mixture of Se-NPs and EPA + DHA at 0.4 and 0.2% with or without exposure to As + T in comparison to other treatments. Results revealed that supplemented group of Se-NPs and EPA + DHA at 0.6% has not much potential to improve the total immunoglobulin and MPO in *P. hypophthalmus*.

#### Concurrent exposure to arsenic and temperature elicits secondary stress response (lipid profiling) but alleviate by dietary EPA + DHA and Se-NPs in *P. hypophthalmus*

Data pertaining to lipid profiling (total lipid, cholesterol, phospholipid, triglyceride and very low density lipoprotein, VLDL) of *P. hypophthalmus* reared in control or under arsenic and high temperature (As + T) are recorded in Table 6. Total lipid, cholesterol, phospholipid and VLDL were noticeably higher (p < 0.01) with from exposeure to As + T and when fed with control diet in comparison to control diet group. However, results of total lipid, cholesterol, triglyceride and VLDL in group fed to Se-NPs and EPA + DHA at 0.6% diet group without and not exposed to stressors (As + T) were similar with control group. Total lipid, cholesterol (p = 0.0073) and phospholipid (p = 0.039) were significantly reduced by supplementation of Se-NPs and EPA + DHA at 0.4 and 0.2% diet groups with or without exposure to As + T in comparison to other treatment groups. Whereas, VLDL (p = 0.024) and triglyceride (p = 0.018) were significantly reduced (p = 0.018) with combinatorial mixture of Se-NPs and EPA + DHA at 0.4% in comparison to fed group of 0.2 and 0.4% EPA + DHA with or without stressors (As + T).

**Table 6:** Mitigation of secondary stress response (total lipid, cholesterol, phospholipid, triglyceride and VLDL) through dietary supplementation of selenium nanoparticles, eicosapentanoic acid and dicosahexanoic acid fed to *P. hypophthalmus* reared in control or under arsenic and high temperature for 105 days.

Treatments	Non-stressors	Stressors (arsenic and temperature)	Non-stressors			Stressors (arsenic and temperature)			P-value
Diets	Control	Control	Se-NPs + EPA + DHA-0.2%	Se-NPs + EPA + DHA-0.4%	Se-NPs + EPA + DHA-0.6%	Se-NPs + EPA + DHA-0.2%	Se-NPs + EPA + DHA-0.4%	Se-NPs + EPA + DHA-0.6%	
Total lipid	13.95^c^ ± 0.91	20.42^d^ ± 0.42	9.02^b^ ± 0.28	6.97^a^ ± 0.52	15.08^c^ ± 0.71	10.42^b^ ± 0.39	8.23^ab^ ± 0.15	19.59^d^ ± 0.60	0.0067
Cholesterol	5.34^c^ ± 0.20	7.01^d^ ± 0.03	3.89^b^ ± 0.18	2.59^a^ ± 0.14	5.77^c^ ± 0.10	4.04^b^ ± 0.12	2.84^a^ ± 0.08	7.10^d^ ± 0.11	0.0073
Phospholipid	4.72^b^ ± 0.25	5.93^c^ ± 0.31	3.60^a^ ± 0.05	3.49^a^ ± 0.21	5.99^c^ ± 0.15	4.94^b^ ± 0.29	3.78^a^ ± 0.18	6.08^c^ ± 0.20	0.039
Triglyceride	3.88^c^ ± 1.26	7.49^d^ ± 0.13	1.53^b^ ± 0.22	0.88^a^ ± 0.52	3.31^c^ ± 0.80	1.43^b^ ± 0.09	1.61^b^ ± 0.12	6.41^d^ ± 0.55	0.018
VLDL	0.78^c^ ± 0.25	1.50^d^ ± 0.03	0.30^b^ ± 0.02	0.18^a^ ± 0.04	0.66^c^ ± 0.10	0.29^b^ ± 0.16	0.32^b^ ± 0.02	1.28^d^ ± 0.11	0.024

Values in the same row with different superscript (a, b, c, d) differ significantly. Data expressed as Mean ± SE (n = 3). Total lipid; cholesterol; phospholipid; triglyceride; very low density lipoprotein (VLDL) mg dL^−1^.

### Concurrent exposure to arsenic and temperature elicits tertiary stress response (growth performance) but alleviate by dietary EPA + DHA and Se-NPs in *P. hypophthalmus*

Data pertaining to growth performance (final weight gain %, FCR, PER, SGR, DGI, TGC and RFI) of *P. hypophthalmus* fed with control and supplemented diets (Se-NPs and EPA + DHA) reared in control or under As + T are recorded in Table 7. Final body weight gain (%), specific growth rate (SGR), protein efficiency ratio (PER), daily growth index (DGI), thermal growth coefficient (TGC) and relative feed intake (RFI) of *P. hypophthalmus* were noticeably inhibited (p < 0.01) by concurrent exposure to As + T and fed with control diet in comparison to control group. Whereas FCR (p = 0.016) was significantly enhanced with concurrent exposure to As + T and fed with control diet in comparison to unexposed group (control group). SGR (p = 0.0078), DGI (p = 0.0043), TGC (p = 0.012) and RFI (p = 0.0038) were noticeably increased in group fed with combinatorial mixture of Se-NPs and EPA + DHA at 0.4 and 0.2% diet group with or without exposure to As + T in comparison to unexposed group (control), stressors group (As + T) and Se-NPs and EPA + DHA at 0.6% diet group. Similarly, final body weight gain (%) (p = 0.029) and PER (p = 0.031) were remarkably increased with Se-NPs and EPA + DHA at 0.4% supplemented diet, in comparison to other treatments, regardless of exposure to stressors. The FCR (p = 0.016) was significantly lower with combinatorial mixture of Se-NPs and EPA + DHA at 0.4% diet group in comparison to other treatments. The group fed with combinatorial mixture of Se-NPs and EPA + DHA at 0.6% showed similar final body weight gain (%), PER, DGI, TGC and RFI with control diet group (unexposed to As + T). Overall results revealed that growth performance of the *P. hypophthalmus* was drastically reduced by exposure to As + T and fed with control diet.

**Table 7:** Mitigation of tertairy stress response (final body weight gain, FCR, PER, DGI, TGC and RFI) through dietary supplementation of selenium nanoparticles, eicosapentanoic acid and dicosahexanoic acid fed to *P. hypophthalmus* reared in control or under arsenic and high temperature for 105 days.

Treatments	Non-stressors	Stressors (arsenic and temperature)	Non-stressors			Stressors (arsenic and temperature)			P-value
Diets	Control	Control	Se-NPs + EPA + DHA-0.2%	Se-NPs + EPA + DHA-0.4%	Se-NPs + EPA + DHA-0.6%	Se-NPs + EPA + DHA-0.2%	Se-NPs + EPA + DHA-0.4%	Se-NPs + EPA + DHA-0.6%	
Final body weight gain %	95.09^b^ ± 5.73	73.67^a^ ± 6.62	127.01^c^ ± 9.35	215.04^e^ ± 11.61	102.51^bc^ ± 4.37	123.33^c^ ± 13.37	199.50^d^ ± 1.15	96.80^b^ ± 8.11	0.029
FCR	3.39^c^ ± 0.13	4.16^d^ ± 0.28	2.88^b^ ± 0.15	2.08^a^ ± 0.08	3.24^c^ ± 0.13	3.02^bc^ ± 0.23	2.18^a^ ± 0.04	3.46^c^ ± 0.23	0.016
SGR	0.68^b^ ± 0.04	0.57^a^ ± 0.02	0.87^ cd^ ± 0.03	1.16^d^ ± 0.02	0.72^c^ ± 0.03	0.86^ cd^ ± 0.07	1.10^d^ ± 0.03	0.68^b^ ± 0.02	0.0078
PER	0.85^b^ ± 0.04	0.78^a^ ± 0.05	1.08^c^ ± 0.03	1.51^e^ ± 0.05	0.96^b^ ± 0.06	1.06^c^ ± 0.09	1.40^d^ ± 0.02	0.90^b^ ± 0.05	0.031
DGI (%)	0.92^b^ ± 0.03	0.74^a^ ± 0.05	1.12^c^ ± 0.05	1.61^d^ ± 0.04	0.92^b^ ± 0.04	1.13^c^ ± 0.09	1.59^d^ ± 0.01	0.93^b^ ± 0.06	0.0043
TGC	0.0396^c^ ± 0.0008	0.0307^a^ ± 0.0005	0.0390^c^ ± 0.0003	0.0388^c^ ± 0.0002	0.0391^c^ ± 0.0005	0.0297^b^ ± 0.0001	0.0307^c^ ± 0.0001	0.0305^c^ ± 0.0002	0.012
RFI	321.12^b^ ± 7.73	303.06^a^ ± 7.12	363.07^c^ ± 9.30	444.58^d^ ± 7.91	330.88^b^ ± 3.09	366.09^c^ ± 14.99	434.19^d^ ± 7.99	331.32^b^ ± 7.58	0.0038

Values in the same row with different superscript (a, b, c, d, e) differ significantly. Data expressed as Mean ± SE (n = 3). FCR: feed conversion ratio; SGR: specific growth rate; PER: protein efficiency ratio; DGI: Daily growth index; TGC: Thermal growth coefficient; RFI: relative feed intake.

### Concurrent exposure to arsenic and temperature elicits tertiary stress response (Bacterial challenges, relative survival (%) and cumulative mortality) but alleviate by dietary EPA + DHA and Se-NPs in *P. hypophthalmus*

**Figure 5:**
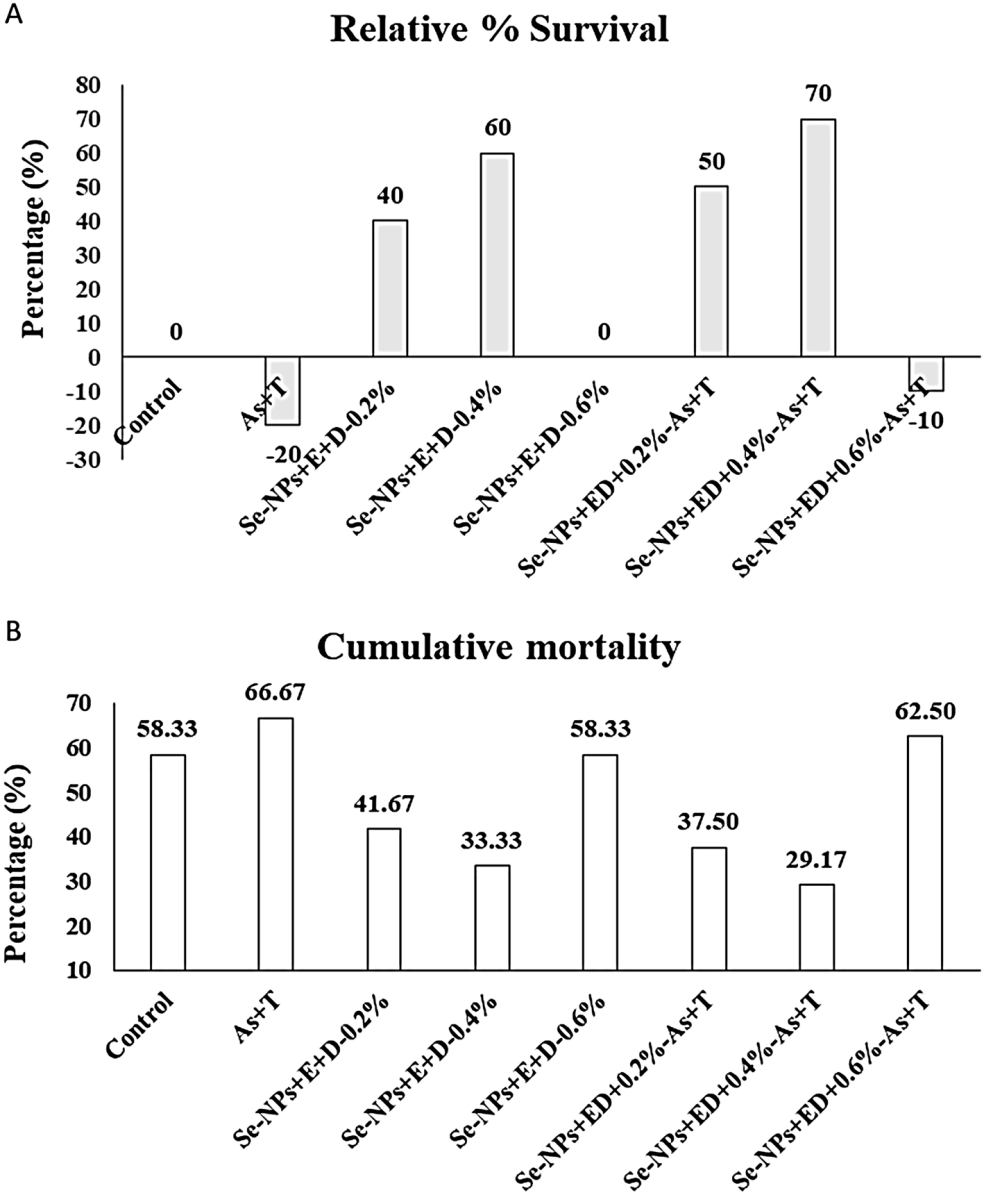
(**A**,**B**) Mitigation of tertiary stress response (relative survival (%) and cumulative mortality) through dietary supplementation of selenium nanoparticles, eicosapentanoic acid and dicosahexanoic acid fed to *P. hypophthalmus* reared in control or under arsenic and high temperature (As + T) for 105 days. Data expressed as Mean ± SE (n = 30).

The results of relative survival (%) (RPS) and cumulative mortality (%) of *P. hypophthalmus* reared in control or under As + T and injected with *A. hydrophila* after experimental period of 105 days are presented in Fig. 5A, B. A 70 and 60% higher RPS was observed in groups fed with Se-NPs and 0.4% EPA + DHA, either in combination with As + T stress or absence of it, respectively. Whereas, RPS of 40 and 50% was observed in groups fed with Se-NPs and EPA + DHA at 0.2% with or without exposure to stressors (As + T), respectively. RPA in the groups exposed to As + T stress and fed with control diet or combination of Se-NPs and 0.6% EPA + DHA was observed lowest; 10 and 20%, respectively. Correspondingly, the cumulative mortality was also highest (66.66 and 62.5%, respectively) in the treatment groups exposed to As + T and fed with control diet or 0.6% EPA + DHA-based diet. The lowest cumulative mortality (%) was observed in the group treated with Se-NPs and EPA + DHA at 0.2 or 0.4% as 41.67 and 33.33% respectively, whereas in the same diet group (0.2 or 0.4% EPA + DHA), with exposure to As + T, the cumulative mortality was observed as 37.20 and 29.17%, respectively.

### Bioaccumulation of arsenic and selenium in fish tissues and water

The concentration of arsenic in water was significantly higher in group treated with As + T and fed with control diet (868.47 µg L^−1^) and EPA + DHA at 0.6% group (774.05 µg L^−1^), in comparison to control and other groups. The lowest arsenic concentration was determined in the group fed with EPA + DHA at 0.4, 0.6 and 0.2% and control group (unexposed group) (Table 8). The bioaccumulation of the arsenic was higher in kidney > liver > gill > muscle > brain. The arsenic bioaccumulation was noticeably reduced with group fed with combinatorial mixture of Se-NPs and EPA + DHA at 0.4 and 0.2% in comparison to other groups. The selenium bioaccumulation in muscle was significantly higher (p < 0.01) in group fed with Se-NPs and EPA + DHA at 0.4% without As + T in comparison to all other experimental groups (Table 7). Similarly, significantly lower (p < 0.01) Se bioaccumulation was found in the group fed with Se-NPs and EPA + DHA at 0.6% regardless of exposure to stressors, in EPA + DHA at 0.4% diet group exposed to stressors and in group fed EPA + DHA at 0.2% regardless to exposure to stressors (Table 8).

**Table 8:** Mitigation of tertiary stress response (residue accumulation of arsenic) through dietary supplementation of selenium nanoparticles, eicosapentanoic acid and dicosahexanoic acid fed to *P. hypophthalmus* reared in control or under arsenic and high temperature for 105 days.

Treatments	Non-stressors	Stressors (arsenic and temperature)	Non-stressors			Stressors (arsenic and temperature)		
Diets	Control	Control	Se-NPs + EPA + DHA-0.2%	Se-NPs + EPA + DHA-0.4%	Se-NPs + EPA + DHA-0.6%	Se-NPs + EPA + DHA-0.2%	Se-NPs + EPA + DHA-0.4%	Se-NPs + EPA + DHA-0.6%
Water (µg L^−1^)	2.56^b^ ± 0.39	868.47f. ± 32.05	2.91^b^ ± 0.43	1.59^a^ ± 0.17	1.12^a^ ± 0.07	513.54^d^ ± 57.24	326.72^c^ ± 47.32	774.05^e^ ± 51.02
Liver (mg kg^−1^)	0.09^a^ ± 0.01	6.42^c^ ± 0.52	0.02^a^ ± 0.01	0.04^a^ ± 0.01	0.02^a^ ± 0.01	0.13^b^ ± 0.01	0.07^a^ ± 0.02	0.11^b^ ± 0.01
Muscle (mg kg^−1^)	0.02^a^ ± 0.0033	1.93^c^ ± 0.31	0.01^a^ ± 0.001	0.02^a^ ± 0.001	0.03^a^ ± 0.007	0.09^b^ ± 0.005	0.08^b^ ± 0.004	0.09^b^ ± 0.003
Gill (mg kg^−1^)	0.05^a^ ± 0.02	1.98^c^ ± 0.41	0.04^a^ ± 0.001	0.05^a^ ± 0.02	0.09^a^ ± 0.01	0.17^b^ ± 0.06	0.14^b^ ± 0.01	0.20^b^ ± 0.02
Kidney (mg kg^−1^)	0.19^a^ ± 0.05	7.98^c^ ± 0.43	0.10^a^ ± 0.001	0.10^a^ ± 0.04	0.20^a^ ± 0.06	0.46^b^ ± 0.001	0.10^a^ ± 0.001	0.52^b^ ± 0.09
Brain (mg kg^−1^)	0.015^a^ ± 0.003	1.23^c^ ± 0.002	0.013^a^ ± 0.001	0.014^a^ ± 0.002	0.087^c^ ± 0.004	0.059^b^ ± 0.003	0.012^a^ ± 0.001	0.090^c^ ± 0.002
Muscle-Se (mg kg^−1^)	0.72^c^ ± 0.05	0.75^c^ ± 0.04	0.64^b^ ± 0.12	0.92^d^ ± 0.02	0.31^a^ ± 0.03	0.74^c^ ± 0.05	0.56^ab^ ± 0.09	0.40^a^ ± 0.03

Values in the same row with different superscript (a, b, c, d, e) differ significantly p < 0.05; Data expressed as Mean ± SE (n = 3).

### Mineral profiling of experimental diets

Data pertaining to mineral profiling of experimental diets is recorded in Table 9. Li, Na, Mg, K, Ca, V, Cr, Mn, Fe, Co, Ni, Cu, Zn, Ga, As and Mo was determined in the experimental diets. Li concentration varies from 0.47 to 0.93, Na 452.62–642.81, Mg 368.518.67, K 719.56–802, Ca 642–845, V 0.024–0.047, Cr 0.89–1.56, Mn 4.18–4.28, Mn 3.45–4.63, Fe 98.65–132, Co 0.21–0.42, Ni 0.47–0.78, Cu 1.85–2.37, Zn 5.89–7.18, Ga 0.26–0.62, As 0.12–0.24, Ru 0.75–1.01, and Mo 0.19–0.86 mg kg^−1^ of diet.

**Table 9:** Mineral profiling of experimental diets.

Elements (mg kg^−1^)	Experimental diets			
	Control	Se-NPs + EPA + DHA-0.2%	Se-NPs + EPA + DHA-0.4%	Se-NPs + EPA + DHA-0.6%
Lithium	0.47 ± 0.03	0.63 ± 0.08	0.74 ± 0.04	0.93 ± 0.07
Sodium	452.65 ± 2.85	523.63 ± 4.17	485.65 ± 6.73	642.81 ± 9.79
Magnesium	368.45 ± 12.56	418.47 ± 11.81	437.82 ± 5.86	518.67 ± 14.85
Potassium	719.56 ± 11.12	813.56 ± 12.51	756.89 ± 7.13	802.65 ± 11.82
Calcium	642.56 ± 17.52	845.65 ± 15.28	784.56 ± 14.75	815.81 ± 17.69
Vanadium	0.047 ± 0.002	0.038 ± 0.004	0.024 ± 0.001	0.031 ± 0.006
Chromium	1.56 ± 0.08	0.89 ± 0.08	1.05 ± 0.081	0.92 ± 0.12
Manganese	4.28 ± 0.79	3.45 ± 0.29	4.63 ± 0.53	4.18 ± 0.038
Iron	114.56 ± 1.67	98.65 ± 2.43	124.83 ± 4.13	132.74 ± 4.21
Cobalt	0.38 ± 0.07	0.21 ± 0.03	0.42 ± 0.08	0.29 ± 0.036
Nickel	0.78 ± 0.03	0.47 ± 0.04	0.69 ± 0.036	0.71 ± 0.04
Copper	2.18 ± 0.07	1.85 ± 0.41	2.09 ± 0.074	2.37 ± 0.058
Zinc	5.89 ± 0.68	6.85 ± 1.28	7.18 ± 0.123	6.43 ± 0.28
Gallium	0.26 ± 0.04	0.38 ± 0.02	0.62 ± 0.045	0.41 ± 0.02
Arsenic	0.18 ± 0.01	0.24 ± 0.04	0.12 ± 0.06	0.16 ± 0.03
Rubidium	0.75 ± 0.03	0.92 ± 0.06	0.87 ± 0.017	1.01 ± 0.07
Molybdenum	0.43 ± 0.02	0.19 ± 0.042	0.37 ± 0.02	0.86 ± 0.06

Data expressed as Mean ± SE (n = 3). Unit: mg kg^−1^ of diet.

## Discussion

Climate change and arsenic pollution are the major challenges for the aquatic ecosystem, which may interfere with the production cycle of the aquatic environment. The arsenic contaminated fish may cause diseases, including cancer in human. The present investigation deals with dietary intervention using combinatorial mixture of Se-NPs at 0.2 mg kg^−1^ diet and eicosapentaenoic acid (EPA) and docosahexaenoic acid (DHA) at 0.2, 0.4 or 0.6%, to mitigate arsenic pollution and temperature induced stress in *P. hypophthalmus*. The results and their discussion are categorized based on the hierarchy of animal stress response as primary, secondary and tertiary.

The primary stress response as cortisol was elevated in fish exposed to arsenic and elevated temperature (As + T). Cortisol is an endocrine hormone secreted from interrenal cells of fish kidney, which is regulated by corticotropin-releasing hormone (CRH)^69^. It can be used as an indicator of fish health due to its role in metabolism, immunity and osmoregulation. Cortisol mobilizes free fatty acid, glucose and amino acid to meet the immediate energy demands of the animal, however, excess mobilization of these metabolites by cortisol reduced the body and muscle mass due to increased energy expenditures^70,71^. Arsenic can target several sites on hypothalamus-pituitary-interrenal axis which might be the reason for increased cortisol secretion in this study. Arsenic can alter the ACTH and effect blood cortisol levels in fish^3,35,72^. In the current study, the cortisol was remarkably reduced with combinatorial mixture of Se-NPs and EPA + DHA at 0.4 and 0.2% diet. Se-NPs have very important role in synthesis of thioredoxin reductase, glutathione peroxidase and deiodinase^73^. It has also functional role in stimulation of adrenocorticotropic hormone (ACTH) which then bind with the steroidogenic membrane receptor to activate the second messenger pathway (cAMP)^74^. Similarly, EPA + DHA are also essential for activation and feedback mechanism of HPA-axis which affects the gluco-corticoid receptor function and transports of the cortisol across the blood brain barrier. EPA and DHA also influence on secretion of corticotrophin releasing hormone^75^. However, EPA + DHA along with Se-NPs are important for cortisol regulation in fish and have role in stress mitigation^76^ and modulatory effect on HPA activity. It is also reported that deficiency of EPA + DHA could increase corticotrophin releasing factor (CRF) and cortisol levels^77^. In the present investigation, the higher level of EPA + DHA at 0.6% diet along with Se-NPs enhanced the cortisol level and revealed that higher inclusion of EPA + DHA induces stress in fish.

Brain work like machineries to control oxidative challenges and if it fails the heat shock protein (HSP) is upregulated to provide the protection against the stress^78^. HSP are multifaceted and can be expressed during a variety of stress including metal pollution^79,80^ and thermal stress^39,81^ and therefore serves as a biomarker for cellular stress response. HSPs comprise a highly conserved, ubiquitously stress-response proteins and function like molecular chaperones which have role in synthesis, protein folding, transport and translocalization of protein and prevent protein aggregation^78^. In the present investigation the supplementation of Se-NPs and EPA + DHA at 0.4 and 0.2% diet significantly reduced the HSP 70 in liver and gill. Se-NPs have essential role in passing the signal from peptides to antigen cells and have their own function such as seleno-methionine. It has role in regulation of HSP during stress condition^4,35^. Data from present study suggested that Se-NPs and EPA + DHA at 0.4 or 0.2% might also be helping in synthesis for maintenance of the cellular homeostasis through correct folding of nascent and stress-accumulated misfolded proteins in the cell^82,83^.

Oxidative stress enzymes viz. CAT, SOD, GST and GPx were elevated after exposure to arsenic and high temperature. Arsenic interacts with cellular antioxidant system and induce oxidative stress resulting in higher inflammation rate and finally accumulates free radicals in the cell^41^. Further, arsenic can also induce generation of reactive oxygen species such as hydroxyl radical (^·^OH), hydrogen peroxide (H_2_O_2_), superoxide anion (O_2_^·−^), peroxyl radicals and singlet oxygen (^−^O_2_). The primary reactive oxygen species (ROS) formed by superoxide anions through metabolic process and or after oxygen activation, can further directly interact with enzymes or generate secondary ROS through metal catalyzed process^84^. ROS can be also formed by three other sources viz. (1) Arsenic reacts with acid and forms arsine which produce large number of free radicals^85^. (2) Methylated can formed redox-active iron from ferritin which has important role in generating oxygen species through conversion of O_2_^·−^ and H_2_O_2_ into the highly reactive ^·^OH radical^86^. (3) Generated during oxidation of arsenite to arsenate^87^. The oxidative stress induced by arsenic and temperature was significantly reduced with combinatorial mixture of Se-NPs and EPA + DHA at 0.4 and 0.2%. Se-NPs and EPA + DHA acts as strong antioxidant which reduced the oxidative stress and enhanced anti-oxidative status of the fish^22,88^. Glutathione peroxidase is the important selenoprotein and shows anti-oxidative activity through selenocysteine which protect cell against oxidative stress and injuries^89^. EPA has an important role in production of eicosanoids and its metabolites improve cellular anti-oxidative mechanisms, which could have resulted in reduced ROS generation against arsenic and high temperature. They also reduce the 8-isoprostane levels and ease oxidative stress^90^. EPA and DHA also have major function in free radical metabolism by activating peroxisome proliferator-activated receptors (PPARs) which have role in neutralization of oxidative stress^91^. At higher level of EPA + DHA (0.6%), elevated oxidative stress in fish as revealed by our present investigation. The present study showed that higher dose of EPA + DHA at 0.6% enhanced the oxidative stress which might be due to production of excessive reactive oxygen species (ROS)^92,93^. A similar result was reported by Todorcevic et al.^93^, that supplementation of higher n-3 HUFAs (High unsaturated fatty acid) enhanced the oxidative stress and apoptosis in *Salmo solar* (Atlantic salmon). However, HUFAs also reduce the fatty acid β-oxidation capacity which facilitates oxidative stress.

In the present investigation, lipid peroxidation (LPO) in liver, gill and kidney were significantly elevated with exposure to As + T, when fed with basal diet or the diet containing higher (0.6%) dose of EPA + DHA. The elevated LPO might be due to production of free radical leading to oxidative damage of PUFAs of cell membranes^94^. Increased LPO was perhaps resultant of free radical mediated damage, including direct reaction with free radicals and deactivation of anti-oxidative mechanisms, as evident from the increased activities of CAT, SOD, GST and GPx in the present study^95^. The application of combinatorial mixture of Se-NPs and EPA + DHA at 0.4% diet reduced the level of LPO which might be due to role of Se in defence mechanism of tissues and organs through GPx and other seleno-enzymes^96^. EPA + DHA at 0.2% also reduced the LPO level in tissues which might be due to efficient anti-oxidative function of EPA and DHA^97^.

Acetylcholine esterase (AChE) is the cholinergic enzyme that breaks down acetylcholine into acetic acid and choline. The AChE activity is mostly present in the brain and to some extent in the muscle tissue. Generally, AChE hydrolyses, acetylcholine into choline and acetate at the postsynaptic membrane from acetylcholine receptors. Therefore, it is mainly found in the neuromuscular junctions and cholinergic synapses in the central nervous system. Hence, it is essential for central and peripheral nervous system for its proper functioning^98^. In the present investigation, AChE was inhibited by concurrent exposure to As + T in fish fed with control diet and higher inclusion levels of EPA + DHA at 0.6% diet. However, the AChE activity was augmented by combinatorial mixture of Se-NPs with EPA + DHA at 0.4 or 0.2% diet, in both brain and muscle tissues. The augmentation of AChE by Se-NPs and EPA + DHA at 0.2 and 0.4% diet might be due to role of selenium in maintenance of central nervous systems as selenoprotein which arise from cortical and hippocampal neurons of brain^99^. EPA has capacity to control the regulation of AChE due to change of neuron membrane fluidity and viscosity, mainly at neuronal synapses^100^.

Vitamin C in the present study was elevated by dietary Se-NPs and EPA + DHA at 0.4 or 0.2% diet group. However, exposure to arsenic and high temperature with basal diet or 0.6% EPA + DHA failed to maintain the vitamin C level in brain and muscle tissues. Vitamin C is potent anti-oxidant needed for collagen synthesis^101^ and has crucial role in metabolism of several biomolecules viz. steroids and detoxification of xenobiotics. Selenium is known to spare vitamin C due to anti-oxidative activities of selenoenzymes which can help in regeneration of ascorbic acid from dehydroascorbic acid. This could be the reason behind the maintained the high level of vitamin C in the tissues^102^.

Immunological attributes viz. total protein, albumin, globulin, A:G ratio, nitro blue tetrazolium (NBT), blood glucose, total immunoglobulin and myeloperoxidase are indicators of better immunity in animal including fish. In the present investigation, arsenic and high temperature had a detrimental effect on the immunological parameters but administration of dietary Se-NPs with EPA + DHA at 0.4 or 0.2% diet reasonably improved immune parameters. Reduced non-specific immunity in the fish observed in the current study could be due to the stress induce by As + T exposure and also from higher inclusion of EPA + DHA at 0.6% diet which lead to non-specific immuno-suppression in fish. Se-NPs are nutritionally important and have activities similar to selenite, methyl selenocysteine and selenomethionine. Although the bioavailability of Se-NPs is higher which cause upregulation of selenoproteins and lower toxicity in animals^40^. Total protein, albumin and globulin are the important parts of nonspecific immunity. Heightened immune response is often correlated with accelerated protein synthesis and was also evident in the current study where Se-NPs and EPA + DHA increased serum protein and the immunity of the fish reared under As + T. Due to nutritional enrichment of the diets, the B-lymphocytes play an important role in enhancement of immunity in fish^103^.

Albumin is needed for transportation of metal, hormones, vitamin, drug, bilirubin, fat metabolites and regulates the free available hormones^103^. Albumin is mainly secreted from liver in fish to fulfil the high energy demand during stress through protein synthesis. In the present investigation, the albumin was less than globulin, however rapid utilization of albumin to meet the immediate energy demand could also increases its rate of synthesis in the liver.

Further, it was also revealed that fish with low globulin is more susceptible to water pollution due to poor immune resistance^104^. Furthermore, nitro blue tetrazolium (NBT) test estimates the functioning of phagocytes, and higher NBT indicates superior non-specific immunity^105^. However, total immunoglobulin is the indictor for strong immunity against microbes and pathogen. It also has role in prevention of tissues damage and proliferation of infectious agent^106^. Cortisol also has depressive role in immunity of the fish. Study conducted by Esteban et al.^107^ revealed that higher cortisol level reduced the immunity of the fish. This claim was also revealed by Gamperl et al.^108^ that deleterious effects of stress on the immune system have been attributed mainly due to elevated levels of cortisol. The depressive effect of cortisol on immune response in fish depend upon both dosage and incubation time.

Myeloperoxidase (MPO) is also crucial for strong immunity. It has role during respiratory burst and forms hypochlorous acid from hydrogen peroxide^109^. The hypochlorous acid is a strong oxidant with cytotoxic effects. In the present study the combinatorial mixture of Se-NPs and EPA + DHA improved MPO level, suggesting their role in increased levels of neutrophils and the control the tissues damaged by other abiotic factors. MPO uses O_2_ derived species (H_2_O_2_) released by neutrophils to oxidize Cl^−^ ions and form HOCl which is important for bactericidal activity^110^. The present study also demonstrated that higher intake of EPA + DHA altered the immunity of the fish. It can also enhance the lipid peroxidation and oxidative stress^111^.

Cortisol, as primary stress response upregulates blood glucose through gluconeogenesis and glycogenolysis pathways^112^. Similarly, the chromaffin cells also enhanced the release of catecholamines, as stress response to increase glycogenolysis and regulate respiratory and cardiovascular function^113^. This process stimulates the glucose to produce enough energy to fulfil the demand. During stress condition, chromaffin cells release adrenaline noradrenaline and catecholamine hormones toward blood circulation which mobilizes cortisol and increases glucose production in fish through glycogenolysis and glucogenesis pathways^112^ to cope with the energy demand produced by the stressor. Moreover, the production of glucose is mostly dependent upon the cortisol, which stimulates liver gluconeogenesis^114^. Finally, the blood glucose released toward the blood circulation enters into cells via insulin action^114^. In the present investigation concurrent exposure to As + T and fed with control diet and Se-NPs and EPA + DHA at 0.6% diet group significantly enhanced blood glucose level. Dietary supplementation of Se-NPs and EPA + DHA at 0.4 and 0.2% reduced blood glucose level could be due to regulate the gluconeogenesis of glucose from protein and amino acid (non-carbohydrate source)^115^. Blood glucose is also correlated with HSP 70, which increases insulin sensitivity and uptake of glucose to meet high energy demands^116^.

Lipid profile viz. total lipid, cholesterol, phospholipid, triglyceride and VLDL was negatively affected by concurrent exposure to As + T, whereas augmented with supplementation of Se-NPs and EPA + DHA at 0.4 and 0.2% diet. The higher total lipid, cholesterol, phospholipid, triglyceride and VLDL were significantly higher with concurrent exposure to As + T and could be due to enhanced lipogenesis by stressors in the fish which is also reflected in the present results of LPO and cortisol. Cortisol also have role in lipolysis and lipogenesis but it is still a matter of debate. The study conducted by Djurhuus et al.^117^ mentions that cortisol could inhibit basal and catecholamine which stimulate lipolysis in cultured human adipocytes, but similar reports in fish are still scanty. Total lipid, cholesterol, phospholipid and VLDL were reduced by selenium supplementation as it has important role in lipid metabolism of the fish^10^. In contrast to our results the triglyceride was enhanced with Se supplementation diet which was reported by Khalil et al.^118^. Selenium deficit diet increases VLDL level in rat which might be due to excessive esterification^119^. Supplementation of dietary PUFAs can reduced the cholesterol level due to enhancement of membrane fluidity, which was reported in gilthead sea bream by Magalhaes et al.^120^.

Growth performance (weight gain %, FCR, PER, TGC, and RFI) comes under tertiary stress response. Results clearly demonstrated that exposure to arsenic and temperature and fed with control diet reduced the growth performance. This is accordance with a previous study where dietary exposure to arsenite impeded growth in rainbow trout (*Oncorhynchus mykiss*)^8^. Se-NPs and EPA + DHA have growth promoter property with smaller concentration. Selenium has very diverse function in metabolism for enzymatic oxidation–reduction and nucleic acid as well as increase protein and water in the cell through oxidizing materials such as carotenoids and vitamin A^121^. Earlier report also suggested that application of dietary EPA + DHA improved growth performance in fish under ideal condition (non-stressors)^118^. In the present investigation, the supplemented groups with 0.2 and 0.4% EPA + DHA have lowered FCR and highest SGR and PER. Hence the weight gain (%) of the fish was higher in all the treatments where EPA + DHA supplemented diets were administered. Luo et al.^122^ had shown on *Acipenser baerii* that EPA and DHA are required for normal growth performance. Similarly, EPA and DHA also helped to improve growth performance in large yellow croaker exposed to biotic stress^20^.

Our previous study on *P. hypophthalamus* fed selenium nanoparticles at 1 mg kg^−1^ diet has also showed improved growth performance^35–37^ in fish. Results of daily growth index, thermal growth coefficient and relative feed intake support the higher body weight gain in the present study. The group supplementation with higher dose of EPA + DHA at 0.6% and Se-NPs at 0.2 mg kg^−1^ did not show improved growth performance in comparison to the control group. Perhaps, higher dose of EPA + DHA at 0.6%, induce oxidative stress made cells more vulnerable to oxidative stress through incorporation of more HUFAs in the cells, leading to compromised growth rate and immunity^123^.

The concentration and bioaccumulation of arsenic in fish tissues revealed that supplementation of Se-NPs and EPA + DHA significantly reduced arsenic bioaccumulation. Se bioaccumulation in muscle tissues was also determined and found that higher Se accumulation in the groups fed with Se-NPs and EPA + DHA at 0.4%. These findings showed that Se have capacity for absorption of arsenic. The study conducted by Moulick et al.^123^ demonstrated that selenium priming of rice seed reduced arsenic bioaccumulation in plants when grown in arsenic contaminated water. Similarly, in the present investigation we found less arsenic in the water in the tanks where Se-NPs supplemented diet was given. This is perhaps due to arsenic absorbing capacity of selenium. EPA + DHA might also aid detoxification of arsenic through liver and kidney as showed in the present investigation^35^. We have also performed the mineral profiling in 17 elements in the experimental diet. The results showed all essential (macro and micro elements) were present adequately in experimental diet.

At the end of experimental trial of 105 days the *P. hypophthalmus* was infected with *A. hydrophila* and cumulative mortality and relative survival (%) (RPS) was observed for 7 days. The present investigation clearly demonstrated that exposure to As + T with basal diet or inclusion of high concentration of EPA + DHA (0.6%) caused higher cumulative mortality and lower RPS. Nevertheless, supplementation of Se-NPs and EPA + DHA with low inclusion level (0.2 and 0.4%) reduced the cumulative mortality and enhanced the RPS. Our previous study on *P. hypophthalmus* reared under multiple stressors and infection with pathogenic bacteria showed that, dietary Se-NPs can reduce the mortality^35,37^. Perhaps, this is due to anti-oxidative and immunomodulatory role Se-NPs through multiple selenoenzymes. The similar results were also reported by Zuo et al.^20^ where DHA/EPA protected the large yellow croaker fish against infestation of parasites.

## Conclusion

The present investigation is the first study to report the role of selenium nanoparticles (Se-NPs) (synthesized from fisheries waste) and eicosapentaenoic acid (EPA) and docosahexaenoic acid (DHA) in mitigation of arsenic pollution, high temperature stress and pathogenic infection in *P. hypophtahlmus*. It also describes the mechanistic role of Se-NPs and EPA + DHA in mitigation of primary stress, secondary stress and tertiary stress response. The growth performance, immune-modulation and most importantly anti-oxidative status was improved by supplementation of Se-NPs at 0.2 mg kg^−1^ and EPA + DHA at 0.4% followed or 0.2%. Overall results also concluded that supplementation of Se-NPs at 0.2 mg kg^−1^ and EPA + DHA at 0.6% is not effective against arsenic pollution and high temperature stress as well as infection against pathogenic bacteria. The combinatorial mixture of Se-NPs and EPA + DHA in the diet may be an efficient feed supplement to develop high nutritive feed against multiple stressors. Therefore, it is recommended that Se-NPs at 0.2 mg kg^−1^ diet and EPA + DHA at 0.4% followed by 0.2% could be included in fish diets for enhancement of growth performance, immunity, anti-oxidative status and RPS (relative percentage survival) as well as reduction of cumulative mortality due to pathogenic bacterial and bioaccumulation of arsenic in critical fish tissues.
